# Multi-Attribute Fusion Algorithm Based on Improved Evidence Theory and Clustering

**DOI:** 10.3390/s19194146

**Published:** 2019-09-25

**Authors:** Wenqing Wang, Yuan Yan, Rundong Zhang, Zhen Wang, Yongqing Fan, Chunjie Yang

**Affiliations:** 1 School of Automation, Xi’an University of Posts and Telecommunications, Xi’an 710121, China; wwq@xupt.edu.cn (W.W.); wangzhen9623@163.com (Z.W.); fanyongqing@xupt.edu.cn (Y.F.); 13772180915@163.com (C.Y.); 2 School of Automation, Central South University, Changsha 410083, China; zrd1018@csu.edu.cn

**Keywords:** data fusion, fuzzy clustering, evidence theory

## Abstract

In most of the application scenarios of industrial control systems, the switching threshold of a device, such as a street light system, is typically set to a fixed value. To meet the requirements for a smart city, it is necessary to set a threshold that is adaptive to different conditions by fusing the multi-attribute observations of the sensors. This paper proposes a multi-attribute fusion algorithm based on fuzzy clustering and improved evidence theory. All of the observations are clustered by fuzzy clustering, where a proper clustering method is chosen, and the improved evidence theory is used to fuse the observations. In the experiments, two-dimensional observations for the street light illumination and for the ambient illumination are used in a campus-intelligent lighting system based on a narrowband Internet of things, and the results demonstrate the effectiveness of the proposed fusion algorithm. The proposed algorithm can be applied to a variety of multi-attribute fusion scenarios.

## 1. Introduction

Multi-attribute fusion is a fusion method involving data from multiple attributes of many sensors, in order to obtain more accurate and reliable conclusions. Many scholars have studied this issue, and have even applied this algorithm to numerous aspects of life. An opinion of multi-attribute information fusion was proposed in order to improve the accuracy of sensor networks. An important part of the unmanned parking-lot system designed by the authors of [1] is license plate recognition. A background fill light is installed under the camera, which can enhance the capture effect of the camera in a dim environment. The main role of the background fill light is to improve the brightness of the capture environment and to make the picture clearer. If the illuminance meter can be used to measure the light source illuminance and the ambient illuminance of the background fill light many times, so as to fuse a switch value, it is possible to accurately determine whether a background fill light needs to be turned on and captured at a certain time. In the literature [2,3,4], the intelligent road-mounting system detected and recognized the license plate, model, color, facial image of the people in the vehicle, and the fill light technology of the camera during capture. The outstanding effect of the LED (Light Emitting Diode) fill light in the work of the authors of [5] showed the growth and development of high-quality vegetable seedlings in low light conditions, light intensities, and photoperiods. It is shown in the literature [6] that an intelligent lighting system for an exhibition hall network, which may be a scene illuminance sensor installed according to the moment of sunlight illumination change, sets a different direct light brightness for exhibits when visitors approach, and also sets up the illumination according to the peak and trough time of the audience entering. Based on multi-dimensional data fusion, a power transformer fault diagnosis system showed the algorithm efficiency in the study [7]. The fuzzy clustering algorithm of the authors of [8] simulated the hydrological process without the relevant data, and better reflected the process of the runoff variation within the error range. The research by the authors of [9] was also prominent. The bias correction term adjusted the influence of initialization on the fuzzy clustering algorithm. 

The above algorithms [1,2,3,4,5,6,7,8] were based on real-datasets and information fusion, and these algorithms assumed, directly or indirectly, that all observations are subject to normal distributions with the same parameter. All of the observations were located near a true value with the same weight. However, because of the inevitable system error of the sensors, the measuring instruments, human factors, and environmental interference, the observations of the sensors are located around several approximate true values; these approximations are considered to be the clustering centers, and different sensors obey normal distributions in a small range around an approximate true value. All of the observations can be clustered according to the deviation of the observations from the true values. Each category can be assigned a different weight. Evidence combination is the indispensable procedure in the improved evidence theory data fusion algorithm. This method can improve the accuracy and credibility of fusion, and this is the role of data fusion. 

The switching threshold of traditional control devices is generally set to a fixed value, which is simple and easy to imply. However, by fusing the data information of multiple attributes to obtain a more precise switching threshold, it is beneficial by saving energy, reducing emissions, and refine the automatic control of the device, promoting the process of a smart city. To this end, based on the idea of fuzzy clustering and evidence theory, this paper proposes a new multi-attribute observation fusion algorithm. The DS evidence theory has steps of grouping the observations before the data fusion. As the fuzzy c-means (FCM) algorithm needs to specify the number of cluster centers in advance, we set the number of cluster centers as two, three, and four, and the data sets were divided into two categories, three categories, and four categories, respectively. The DS evidence theory was used on every clustering result in order to obtain the most accurate data for the fusion results. This data can be considered as the switching threshold of the device for a period of time. This threshold can provide more abundant information than single one-dimensional data, so as to achieve the purpose of improving the fusion precision. The algorithm does not need any prior information or the historical data of the sensors, nor does it need to assume that sensor observations obey the normal distribution of the same parameter [10,11]. This algorithm has the characteristics of a wide application range, simplicity, and a high reliability of the fusion results.

As such problem settings and application scenarios are quite common, the method can also be applied to other scenarios that require multi-attribute data fusion, and two-dimensional attributes are extended to multidimensional attributes, such as traffic control, battlefield situation estimation, target classification and tracking, smart cities, and agriculture.

## 2. Algorithm Description

### 2.1. Fuzzy C-Means Clustering Algorithm

Research on clustering algorithms has achieved many results [12,13]. Comparing the K-means algorithm, hierarchical clustering algorithm, self-organizing maps (SOM) neural network clustering algorithm, and fuzzy c-means (FCM) clustering algorithm, it has been found that although the hierarchical clustering does not need to determine the category number, once a segmentation or merging was performed, it could not be corrected. The clustering quality was limited [14]. The SOM neural network has a longer processing time, because its mapping has a topology-like retention characteristic, similar to the brain nervous system. FCM is a clustering algorithm based on data division; this algorithm was thought to be an improvement to the hard c-means (HCM) algorithm [15]. The FCM algorithm obtains the membership of each sample to all of the category centers by optimizing the objective function. It can determine generics of the samples so as to achieve the purpose of grouping the observations automatically, and its accuracy is higher than the classical K-means clustering algorithm. Therefore, the FCM algorithm was selected for the experiment. 

In this paper, 100 sets of observations with two attributes were tested by measuring the ambient illuminance and light illuminance of street lights, based on a smart campus lighting system. These observations were clustered by an FCM algorithm. The idea of the algorithm has been described in detail [16,17,18,19,20].

It divides 100 sets of observations $x_{n}(n=1,\;2,\ldots ,100)$ into categories. Each sample is not strictly divided into a certain category, but belongs to a category in a certain membership grade. The division can be stated by the membership matrix, $U={\left[u_{ik}\right]}_{a\times 100}$. $u_{ik}$ shows the membership grade of i(i=1, 2,…,100) to k(k=1, 2,…,a). $v=\left(v_{1},\;v_{2},\;\ldots ,\;v_{a}\right)$ is the center of the clustering, and $d_{ik}=∥x_{i}-v_{k}∥$ is the Euclid distance between the samples and each clustering center. FCM divides the objective function as follows:

(1) $$\min J(U,\;v)=\sum_{i=1}^{100}\sum_{k=1}^{a}u_{ik}^{h}d_{ik}^{2}$$

where the parameter h>1 is a weighted index to control the fuzzy degree of the membership matrix, U. h will be fuzzier when it gets bigger. When h=1, the fuzzy clustering will decline to the HCM clustering. 

For the FCM algorithm, the algorithm has to specify the number of categories in advance, initialize *a* and *h* here, a=2, 3, 4 and h=2. $\varepsilon =1\times 10^{-4}$ is the accuracy of the cluster center. The initial cluster centers were generated randomly. The membership matrix can be calculated according to Equation (3). Secondly, we used Equation (2) to adjust the cluster centers and categories. Finally, in the light of the termination condition, we used $\varepsilon =1\times 10^{-4}$ to determine whether the accuracy of the cluster centers met the requirement. We recalculated the membership matrix and cluster centers if the termination condition was not met by the iteration. The clustering effect of the different categories of these 100 samples were different.

Using the Lagrange multiplier method, the necessary conditions to minimize Equation (1) were as follows:

(2) $$u_{ik}={\left(\sum_{j=1}^{a}{\left(\frac{\left\|x_{i}-v_{k}\right\|}{\left\|x_{j}-v_{k}\right\|}\right)}^{\frac{2}{h-1}}\right)}^{-1}$$

(3) $$v_{i}=\frac{\sum_{k=1}^{n}{\left(u_{ik}\right)}^{h}x_{k}}{\sum_{k=1}^{n}{\left(u_{ik}\right)}^{h}}$$

**Figure 1:**
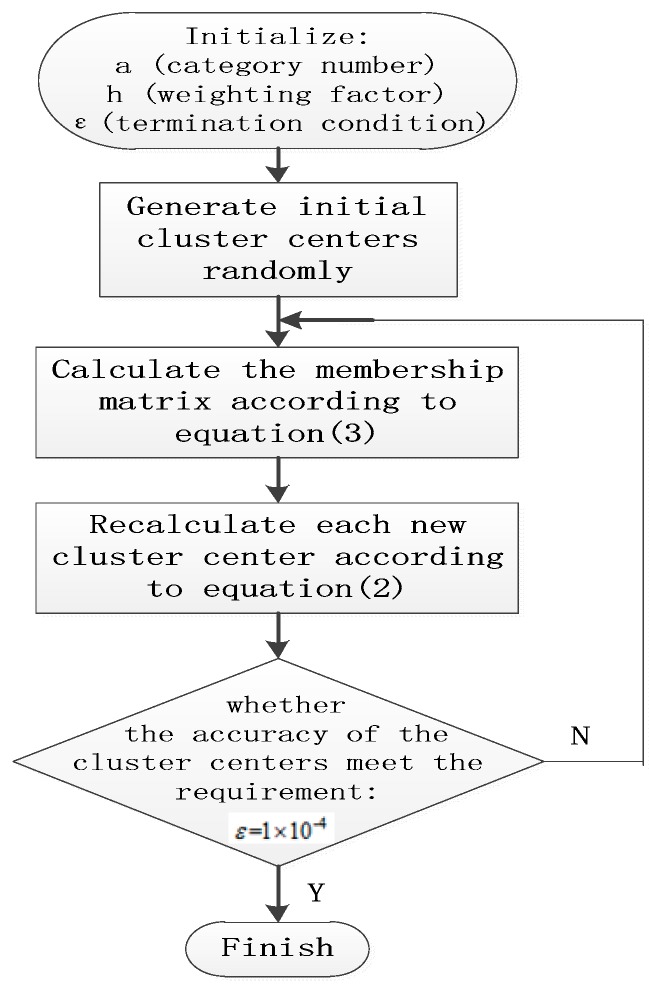
Flow chart of the fuzzy c-means (FCM).

The specific flow chart is shown in Figure 1.

### 2.2. Improved Dempster–Shafer Evidence Theory

The Dempster–Shafer evidence theory (DS evidence theory) was presented by Dempster, and Shafer perfected the theory based on the research of Dempster. The DS evidence theory can describe the uncertainty and incompleteness of the evaluation information well [21].

**Identification framework**. Given a finite nonempty set identification framework, it includes N mutually exclusive elements,$\Theta =\{L_{1,}L_{2,\cdots ,}L_{N}\}$. The set of all of the subsets in Θ is called a power set of Θ, that is, $2^{\Theta }$.

**Basic probability assignment**. Suppose that in the identification framework of Θ, m(L) satisfies the mapping of $2^{\Theta }\to \left[0,\;1\right]$, L is any subset of the identification framework. L makes m(L)>0, which is known as the focal element, and m(L) is regarded as the basic probability assignment (BPA; also known as the mass function), which indicates the degree of support for proposition L. L satisfies the following:

(4) $$\left\{ \begin{array}{l} \sum m(L)=1\\ m(\emptyset )=0 \end{array}\right.$$

We used the obtained mass function to make a decision, then, we used the Euclidean distance to help find the data near the true value, and even to find the true value, eliminate the elements that can be removed, and fuse the evidence with a fusion rule based on the reliability factor.

#### 2.2.1. Judging the Accuracy of the Observations

All of the observations are denoted by $L_{i}=\left(x_{i},y_{i}\right)$, the distance from observation $L_{i}$ to observation $L_{j}$ is set to ${\sqrt{{\left(x_{i}-x_{j}\right)}^{2}+{\left(y_{i}-y_{j}\right)}^{2}}}_{.}$ $d_{i}$ indicated the average value of the distance ${\sqrt{{\left(x_{i}-x_{j}\right)}^{2}+{\left(y_{i}-y_{j}\right)}^{2}}}^{}$ between the two samples. Moreover, $\bar{d}$ representd the mean distance between all of the observations, and the formulas for $d_{i}$ and $\bar{d}$ are as follows:

(5) $$d_{i}=\sum_{i=1}^{N}\sqrt{{\left(x_{i}-x_{j}\right)}^{2}+{\left(y_{i}-y_{j}\right)}^{2}}$$

(6) $$\bar{d}=\;\sum_{n=1}^{N}d_{i}/N$$

Because the observations from the sensors are different, the normal observations in all of the deviation ranges should be distributed near the true value, and the observations with large deviations are far from the normal observations.

**Definition** **1.** $U_{1}$*is a small deviation set and*$U_{2}=\bar{U_{1}}$*is a large deviation set, where*$U_{1}\cup U_{2}=\Theta$. $U_{1}$*meets the following requirements:*

(7) $$\left\{ \begin{array}{l} d_{i}\;<\;\bar{d}\;\left(\forall \;L_{i}\subseteq U_{1}\right)\;\\ d_{i}\;\geq \;\bar{d}\;\left(\forall \;L_{i}⊄U_{1}\right)\; \end{array}\right.$$

#### 2.2.2. Observations Converted to Evidence

Converting observation $L_{i}$ to evidence $e_{i}$ is the core of evidence theory and the basis of data fusion.

**Definition** **2.** *If any of the observations of*$L_{i}$*exist as*$\Delta_{i}\geq 0$*, such that the true value,*$L_{0}$*, is within the neighborhood,*$L_{i}$*of*$\delta_{i}$*(the circle center of*$L_{i}$*and the radius of*$\Delta_{i}$*),*$\delta_{i}$*is the scattering interval of*$L_{i}$*, and*$\Delta_{i}$*is called the scattering radius. The size of*$\Delta_{i}$*is determined by the deviation of the observation*$L_{i}$*from the true value*$L_{0}$.

If $L_{i}\subseteq U_{1}$, $L_{i}$ is a small deviation observation, the scattering radius is relatively small, and $L_{i}$ is located in the $\delta_{1}$ circle (in the center of $L_{i}$, with a radius of $\bar{d}$). There are K observations; $X_{K}\subseteq \Theta \left(K=1,\;2,\;\ldots ,\;K\right)$ are small deviation observations, and it is considered that the K observations are close to the true value, $L_{0}$, with the same probability, that is, the K small deviation observations obtain a basic probability allocation of 1/K, and the basic probability assignment of the remaining N−K observations are 0. The mass function of the evidence, $e_{i_{1}}$, obtained by $L_{i}$, is as follows:

(8) $$m_{i}(X_{K})=1/K(\forall X_{K}\in \delta_{1}).$$

$L_{i}$ is a large deviation observation if $L_{i}\subseteq U_{2}$, which is far away from the true value, $L_{0}$, and if the scattering radius is large, $d_{\max}$ stands for the distance between the maximum and minimum values, and the following formula is obtained:

(9) $$d_{\max}=\max\left\{L_{i}\right\}-\min\left\{L_{i}\right\}.$$

Taking $d_{\max}$ as the scattering radius of $L_{i}$, because the true value, $L_{0}$, must lie in the intermediate of the maximum and the minimum observations, all the observations are included in $\delta_{2}$ (the circle centered $L_{i}$ and radius $d_{\max}$), obtaining the evidence’s ($e_{i_{2}}$) mass function, as follows:

(10) $$m_{i}(X_{K})=1/N\;(\forall L_{i}\subset \Theta ).$$

Then, the evidence, $e_{i_{2}}$, is converted from the large deviation of the observation, $L_{i}$, and each observation obtains a basic probability assignment of 1/N. The process of generating N initial evidence $e_{i}\left(i=1,\;2,\;\ldots ,\;N\right)$ from N observations have been completed [22,23].

The above initial evidences may contain focal points of both $U_{1}$ and $U_{2}$, and the degree of deviation in each observation has not been considered. The initial evidence, $e_{i}$, is corrected as follows:

$\forall \;X_{1},\;X_{2}\in U_{1}$, and the ratio of basic probability assignment obtained by $X_{1}$ and $X_{2}$ is as follows:

(11) $$m_{i}\left(X_{1}\right)/m_{i}\left(X_{2}\right)=d_{X_{2}}/d_{X_{1}}.$$

$\forall \;X_{K}\in U_{1}$ and $\forall \;L_{i}\subseteq U_{2}$, between $X_{K}$ and $L_{i}$, the rate is as follows:

(12) $$m_{i}\left(X_{K}\right)/m_{i}\left(L_{i}\right)=d_{\max}/d_{K}.$$

The above two formulas produce a set of correction coefficients $\left\{\omega_{n}\right\}\left(n=1,\;2,\;\ldots ,\;N\right)$ for the normalized, weighted, and corrected evidence, with the following equation:

(13) $$M_{i}\left(L_{i}\right)=\omega_{i}*m_{i}\left(L_{i}\right)/\sum_{n=1}^{N}\omega_{n}*m_{i}\left(L_{n}\right).$$

#### 2.2.3. Combination of Evidence

There may be a high conflict between all of the evidence obtained by the above algorithm, and in order to avoid the unreasonable weight distribution, combined with the literature [10,11], the evidence fusion formula that assigns the probability of supporting evidence conflict to the observation is as follows:

(14) $$m\left(L_{i}\right)=\prod_{i=1}^{N}M\left(L_{i}\right)+c*\bar{M_{i}}\left(L_{i}\right)$$

where c is the conflict factor and $\bar{M_{i}}\left(L_{i}\right)$ is the average distribution of $L_{i}$ in all of the evidence. The formulas are as follows:

(15) $$c=1-\sum_{i=1}^{N}\prod_{n=1}^{N}M_{i}\left(L_{i}\right)$$

(16) $$\bar{M_{i}}\left(L_{i}\right)=\frac{1}{N}\sum_{i=1}^{N}M_{i}\left(L_{i}\right).$$

The basic probability distribution $m\left(L_{i}\right)$ is the weight obtained by $L_{i}$, and the fusion result is as follows:

(17) $$L_{0}=\sum_{i=1}^{N}L_{i}m\left(L_{i}\right)$$

## 3. Experiment Analysis

In order to verify the effectiveness of this algorithm, 100 sets of two-dimensional datasets were used for the experiments. The datasets involved the ambient illumination and street light illumination of intelligent campus lighting systems. As a result of the measurement error of the sensors and other factors, such as ambient lighting, the ambient illumination and street lights of different streets were measured to be different at the same time, but the observations were always distributed near a certain value. The reference value of the switch threshold is 20.00 LX.

The 100 sets of data were collected within 5 min of nightfall. At dusk, the brightness of the environment changed rapidly, and the process from daylight to dark was more obvious. Therefore, the ambient brightness varied from bright to dark, as the x column of the two-dimensional data sets. When the ambient light level was dimmed from light to dark, the data of this column should be changed from large to small. As the brightening of the street light source is a process, when the street light turned on, the brightness of the light source is relatively small. After a period of time, the light source will be brightened to a normal brightness. Thus, the change in the brightness of the light was recorded within 5 min. The light is a process from dark to light, as seen in the y column of the two-dimensional datasets, and the data of this column should be changed from small to large. This is the data law of the 100 datasets.

By combining the two brightness characteristics of street lights, a street light switching threshold suitable for the specific environment will be obtained. The brightness of the single light should be different in different weather, locations, human flows, and so on. The threshold obtained by fusion is also different. This threshold helps the street light’s manager to manage and control the switch of the street lights precisely. The administrator can achieve precise control of the street light switch in different weather, environments, and seasons, according to the fusion threshold [24].

In this paper, the distance between the two observations was calculated by the Euclidean distance. These two-dimensional observations were clustered by MATLAB software programming. FCM helps to obtain clustering images and clustering conditions. Each clustering observation is regarded as the identification framework, and is then converted into evidence. By modifying and combining the evidence, we get the weight assignment of the observations, which is called the mass function, and the preliminary fusion value of the samples can be obtained. Finally, the data of all of the categories are weighted according to different weights. The fusion result is one-dimensional data. We compared the fusion results of two, three, and four categories, using the highest precision data as the final fusion result, which is the switching threshold of the street light in smart campus lighting system.

The observations are as follows:

X = [24.22,27.45;23.9,27.68;23.72,27.76;23.55,27.83;23.38,27.9;23.22,27.95;23.05,28.11;23,28.21;22.93,28.33;22.89,28.52;22.85,28.54;22.66,28.6;22.6,28.68;22.55,28.69;22.5,28.7;…18.3,34.66;18.21,34.78;18.17,34.82;18.1,34.86;17.98,34.91;17.93,35.02;17.88,35.1;17.8,35.18;17.66,35.27;17.59,35.35;17.51,35.41;17.38,35.64;17.21,35.9;16.9,36.12;16.66,36.34].

**Figure 2:**
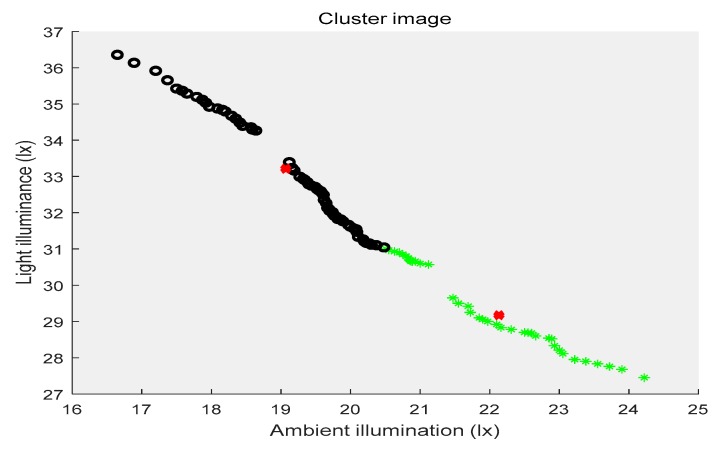
FCM cluster image (two cluster centers).

**Figure 3:**
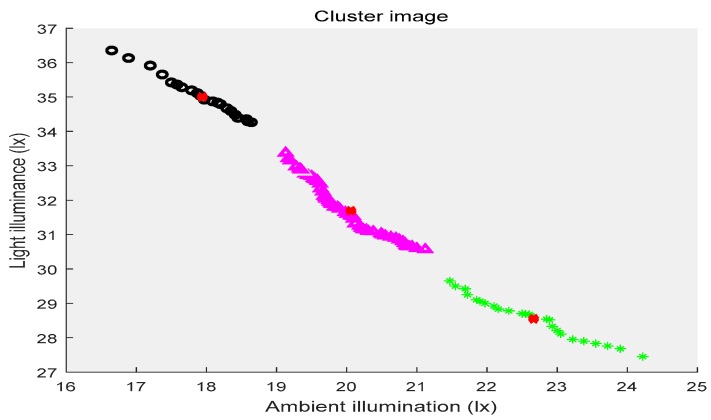
FCM cluster image (three cluster centers).

**Figure 4:**
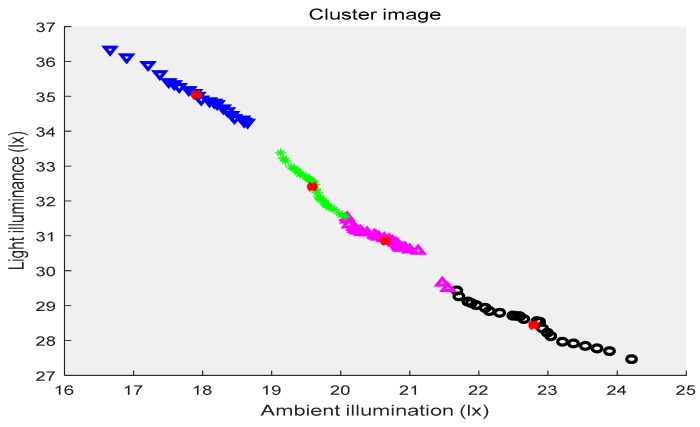
FCM cluster image (four cluster centers).

In the above three figures, the abscissa indicates the ambient illuminance, and the illuminance of the street lights is shown in the ordinate. The samples with two attributes get the corresponding cluster images under the constraints of different cluster centers, respectively. Different categories of samples are represented by different shapes and colors. The red “x” indicates the cluster center. The first category of data is depicted by black circles, the second category is represented by purple triangles, the third category is shown by green ”*”, and the fourth category is presented by blue triangles. Figure 2 shows the location of the cluster center and which category all of the sample points belong to when the number of the cluster centers is two. Figure 3 shows the number of cluster centers set to three, the location of the cluster center and the category to which all of the samples belong. The number of cluster centers is four, and the location of the cluster center and the category to which all of the data belong are shown in Figure 4. For the different datasets, it cannot be decided whether to divide the dataset into several categories in order to achieve the best fusion effect. Therefore, the DS theory was used to fuse the three clustering cases, and then the fusion results were compared. The highest precision result was the threshold of the intelligent campus lighting system.

As can be seen from Table 1, Table 2 and Table 3, the data fusions are firstly performed by each group of data obtained by FCM clustering, and then the fusion results of these three groups are weighted and fused to obtain the final fusion result. When the number of clusters is three, the relative error is 2.003%, the relative error with two categories is 2.431%, and the relative error with four categories is 2.015%, so it is best to divide the dataset into three categories. Take the third group of the highest precision fusion results as an example—the fusion result is shown in Table 4.

The final fusion result of the three groups of data shown in Table 2 is 20.4006, and the relative error is 2.003%.

The fixed threshold method is a global fixed threshold, which means that all of the datasets of a system are binarized with a unified threshold. The multi-attribute fusion algorithm proposed in this paper can fuse a threshold adaptively, and can dynamically change the threshold to reduce the error. Take the data sample of high brightness ambient illumination as an example. The reference value of the switch threshold is 30.85 LX.

**Figure 5:**
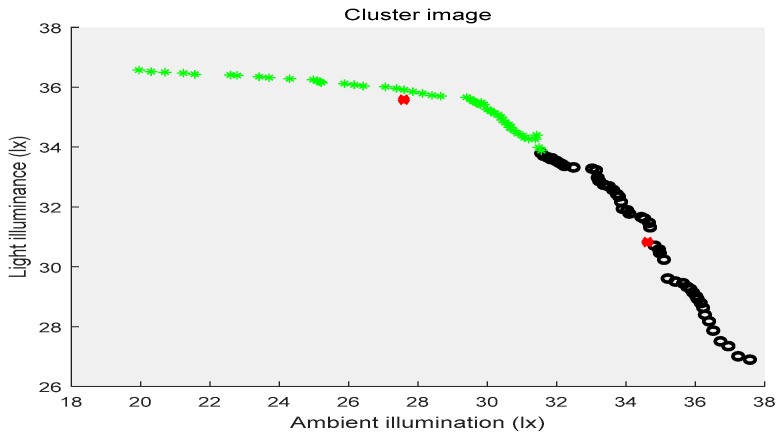
FCM cluster image based on the fixed threshold method (two cluster centers).

**Figure 6:**
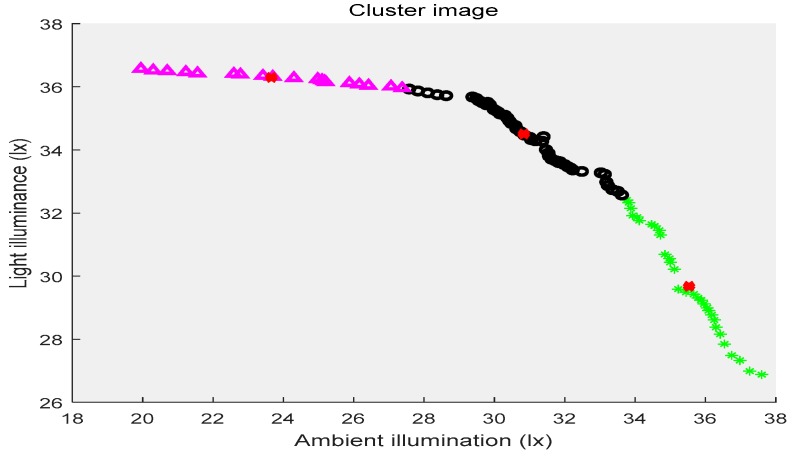
FCM cluster image based on the fixed threshold method (three cluster centers).

**Figure 7:**
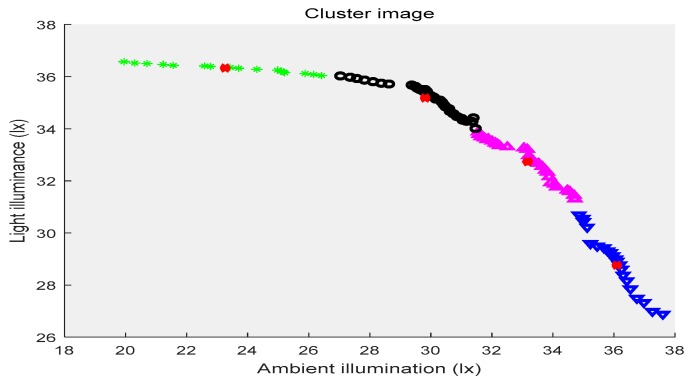
FCM cluster image based on the fixed threshold method (four cluster centers).

Figure 5, Figure 6 and Figure 7 show the cluster centers of different categories. From the following Table 5, Table 6 and Table 7, it can be seen that when the samples are clustered into four categories, the fusion result has the highest precision, and the fusion result is 31.4590. The fixed threshold method aims to set all of the street lights to the same switch threshold. If the switch threshold is still set to 20.00 LX in the place where the ambient brightness is high, a large error will occur, resulting in a waste of energy.

The literature [25] shows us the remediation of failed identification in product multi-source information fusion based on DS evidence theory. This work is aimed at the wear and tear, corrosion, and pollution of two-dimensional data matrix symbols lead by the complexity of the discrete manufacturing enterprise, production environment, and production process. The method firstly established a remedy technical framework. Secondly, it calculated the similarity measurement of the multi-attribute data identification of invalid distinguish goods. Finally, it recognized the data matrix code based on the multi-attribute fusion of DS theory. The experiment analysis demonstrated a processing statistic for 120 failure data matrix codes, and matching the correct rate can be up to 96%. Compared with this algorithm, the correct rate of the algorithm proposed in this paper is above 97.997%, which has the advantage of a higher accuracy. This opinion has more robustness to switching threshold of the smart street light system. This method can better integrate the street light switching threshold to be suitable for a specific environment, which provides effective support for other two-dimensional information fusion application scenarios.

## 4. Conclusions

Based on the FCM clustering algorithm and improved evidence theory, a novel multi-attribute fusion algorithm is proposed. The algorithm can combine the light source illumination and ambient illumination of the smart campus street light system effectively in order to obtain the switching threshold. To the best of our knowledge, it is the first time that the FCM clustering algorithm is being combined with evidence theory for multi-attribute fusion. The effectiveness of the algorithm is also tested by the real-life datasets based on the smart campus street lights system. The effect of the algorithm is proven by the change of the cluster centers, which lays a foundation for future multi-attribute fusion.

This algorithm also involves some future issues. First of all, the clustering method cannot be fully applied to the data of all of the application scenarios. When the number of samples is large, the FCM algorithm needs to define the cluster center, and it easily falls into the local optimal solution. Secondly, is how to distinguish useful information from noise in the process of classifying samples. The next step is to try other algorithms (such as the Bayesian estimation) to cluster the initial observations more accurately. For different application problems, how to make the algorithm adapt to the cluster center intelligently also has a lot of space for improvement.

## Figures and Tables

**Table 1:** Clustering results with two cluster centers.

Group	Data Number	Observations	Result
1	61	X = [24.22,27.45;23.9,27.68;23.72,27.76;23.55,27.83;23.38,27.9; … 19.78,31.9;19.76,31.95;19.75,32;19.71,32.04;19.7,32.08]	21.5649
***2***	39	X = [19.68,32.12;19.66,32.25;19.63,32.34;19.62,32.48;19.59,32.55; … 17.51,35.41;17.38,35.64;17.21,35.9;16.9,36.12;16.66,36.34]	19.0030
Fusion result			20.5658

**Table 2:** Clustering results with three cluster centers.

Group	Data Number	Observations	Result
1	25	X = [24.22,27.45;23.9,27.68;23.72,27.76;23.55,27.83;23.38,27.9; … 21.85,29.1;21.72,29.25;21.69,29.42;21.55,29.5;21.47,29.65]	22.8750
2	54	X = [21.12,30.57;21,30.6;20.93,30.62;20.89,30.65;20.87,30.66;20.86,30.68; … 19.33,32.92;19.28,32.98;19.2,33.15;19.17,33.22;19.13,33.38]	20.1831
3	21	X = [18.65,34.25;18.6,34.28;18.58,34.34;18.46,34.38;18.42,34.47; … 17.51,35.41;17.38,35.64;17.21,35.9;16.9,36.12;16.66,36.34]	18.1422
Fusion result			20.4006

**Table 3:** Clustering results with four cluster centers.

Group	Data Number	Observations	Result
1	23	X = [24.22,27.45;23.9,27.68;23.72,27.76;23.55,27.83;23.38,27.9; … 21.97,29;21.9,29.06;21.85,29.1;21.72,29.25;21.69,29.42]	22.9340
2	30	X = [21.55,29.5;21.47,29.65;21.12,30.57;21,30.6;20.93,30.62; … 20.09,31.53;20.06,31.55;20.01,31.6;19.98,31.65;19.9,31.75]	20.7256
3	26	X = [19.87,31.8;19.83,31.81;19.8,31.88;19.78,31.9;19.76,31.95; … 19.33,32.92;19.28,32.98;19.2,33.15;19.17,33.22;19.13,33.38]	19.6177
4	21	X = [18.65,34.25;18.6,34.28;18.58,34.34;18.46,34.38;18.42,34.47; … 17.51,35.41;17.38,35.64;17.21,35.9;16.9,36.12;16.66,36.34]	18.1422
Fusion result			20.4030

**Table 4:** Group 3 evidence of mass function.

Evidence	Basic Probability Assignment										
	$m_{3}\left(L_{80}\right)$	$m_{3}\left(L_{81}\right)$	$m_{3}\left(L_{82}\right)$	$m_{3}\left(L_{83}\right)$	$m_{3}\left(L_{84}\right)$		$m_{3}\left(L_{96}\right)$	$m_{3}\left(L_{97}\right)$	$m_{3}\left(L_{98}\right)$	$m_{3}\left(L_{99}\right)$	$m_{3}\left(L_{100}\right)$
$e_{80}$	0.0088	0.0261	0.0610	0.1092	0.1757	…	0	0	0	0	0
$e_{81}$	0.0088	0.0261	0.0610	0.1092	0.1757	…	0	0	0	0	0
$e_{82}$	0.0060	0.0177	0.0414	0.0740	0.1192	…	0	0	0	0	0
$e_{83}$	0.0031	0.0093	0.0217	0.0388	0.0624	…	0	0	0	0	0
$e_{84}$	0.0024	0.0093	0.0217	0.0388	0.0624	…	0	0	0	0	0
	…	…	…	…	…	…	…	…	…	…	…
$e_{96}$	0.0017	0.0051	0.0119	0.0212	0.0342	…	0.0070	0.0061	0.0053	0.0046	0.0046
$e_{97}$	0.0017	0.0051	0.0119	0.0212	0.0342	…	0.0070	0.0061	0.0053	0.0046	0.0046
$e_{98}$	0.0017	0.0051	0.0119	0.0212	0.0342	…	0.0070	0.0061	0.0053	0.0046	0.0046
$e_{99}$	0.0017	0.0051	0.0119	0.0212	0.0342	…	0.0070	0.0061	0.0053	0.0046	0.0046
$e_{100}$	0.0017	0.0051	0.0119	0.0212	0.0342	…	0.0070	0.0061	0.0053	0.0046	0.0046
Synthetic evidence	0.0023	0.0070	0.0169	0.0327	0.0522	…	0.0030	0.0026	0.0023	0.0020	0.0020
Fusion result	18.1422										

**Table 5:** Clustering results with two cluster centers based on the fixed threshold method.

Group	Data Number	Observations	Result
1	52	X = [37.6,26.88;37.26,26.99;36.98,27.33;36.75,27.49;36.54,27.85; … 31.58,33.78;31.56,33.88;31.49,33.99;28.13,35.79;31.38,34.26]	34.8223
***2***	48	X = [31.19,34.27;31.07,34.31;31.03,34.38;30.88,34.45;30.76,34.56; … 21.56,36.43;21.23,36.47;20.7,36.5;20.3,36.52;19.95,36.57]	29.2341
Fusion rusult			32.1400

**Table 6:** Clustering results with three cluster centers based on the fixed threshold method.

Group	Data number	Observations	Result
1	50	X = [37.6,26.88;37.26,26.99;36.98,27.33;36.75,27.49;36.54,27.85; … 31.76,33.64;31.65,33.69;31.58,33.78;31.56,33.88;31.49,33.99]	34.9136
2	19	X = [28.13,35.79;31.38,34.26;31.19,34.27;31.07,34.31;31.03,34.38; … 30.12,35.19;30.09,35.21;30.01,35.26;29.89,35.42;29.83,35.49]	30.5002
3	31	X = [29.8,35.41;29.75,35.45;29.68,35.48;29.62,35.52;29.58,35.54; … 21.56,36.43;21.23,36.47;20.7,36.5;20.3,36.52;19.95,36.57]	27.2545
Fusion rusult			31.7007

**Table 7:** Clustering results with four cluster centers based on the fixed threshold method.

Group	Data Number	Observations	Result
1	34	X = [37.6,26.88;37.26,26.99;36.98,27.33;36.75,27.49;36.54,27.85; … 33.82,32.33;33.76,32.41;33.65,32.55;33.53,32.67;33.38,32.72]	35.6463
2	17	X = [33.25,32.85;33.21,32.96;33.16,33.21;33.05,33.26; 32.51,33.3; … 31.65,33.69;31.58,33.78;31.56,33.88;31.49,33.99;31.42,34.4]	32.3908
3	28	X = [31.38,34.26;31.19,34.27;31.07,34.31;31.03,34.38;30.88,34.45; … 29.51,35.61;29.4,35.66;28.65,35.7;28.4,35.73;28.13,35.79]	30.3405
4	21	X = [27.85,35.85;27.6,35.91;27.38,35.96;27.06,36.01;26.42,36.04; … 21.56,36.43;21.23,36.47;20.7,36.5;20.3,36.52;19.95,36.57]	25.4167
Fusion result			31.4590
